# An Inquiry into Dysenteric Bilious Fever

**Published:** 1840

**Authors:** W. K. Bowling

**Affiliations:** Adairville, Ky.


Art. III.—*An inquiry into Dysenteric Bilious Fever.* By W. K. Bowling, M. D., of Adairville, Ky.

The topography of the southern portion of the Green river country renders it peculiarly obnoxious to every variety of miasmatic disease, but especially to dysentery.

That portion of it in which the author resides, presents an extensive plain of many miles, scarcely relieved by the slightest eminence, covered, where not cultivated, with a dense and almost impenetrable growth of dwarf hickory, hazle and sumach, and a young forest of black oak, wild-cherry and sassafras, the product of the last twety years; the country having been originally "*barren.*" It is irrigated and fertilized by the north and middle forks of Red river, which after wending tardily along some twenty miles, almost parallel, and seldom more than five miles apart, unite to pour their common waters into the Cumberland thirty miles below.

For severeal miles north and south of these rivers the soil is strong and productive, but between them it is exceedingly rich, yielding the most luxuriant crops of grain, hemp and tobacco. The fine water power afforded by this river and its tributaries, could not fail to attract capitalists, who have erected large flour manufactoring mills, and cotton factories, within practicable distances, almost from its source to its confluence. The water being thus confined by numerous dams, and large quantities of drift wood and leaves from the small tributaries being deposited at every freshet, in the comparatively still water of the ponds, it follows that an abundant material for the generation of miasm is always on hand. Accordingly about the middle of July in every year, some of the varieties of bilious fever make their appearance, the multiplication of cases being almost in direct ratio with the heat of the weather, and lowness of water in the dams.

Simultaneously with the appearance of bilious fever, cases of dysentery are developed, in the same district, the latter multiplying as the former spreads, and becoming of less frequent occurrence as that diminishes. Not that the dysentery uniformly accompanies, and is associated with bilious fever, for the latter not unfrequently prevails through the summer and autumn, in the total absence of the former. But whenever dysentery *does* appear—and it prevails in this district two seasons out of three—it is always the *accompaniment* of bilious fever.

That the predisponent cause of dysentery is to be sought for in the exhalations from animal and vegetable decomposition, admits of as little doubt, as that bilious remittent and intermittent fever owe their origin to that cause. It is in climates most obnoxious to bilious fever, and at seasons, and in localities favorable to the rapid generation of malaria, that dysentery is rife. Indeed, for a number of years I have regarded dysenteric phenomena as nothing more than adventitious manifestations superadded to the suite of symptoms usually characteristic of bilious fever. How the nervous impressibility in the one case should be so operated upon by the morbific cause as to result in bilious fever, and in the other superinduce dysentery, is, to me, inexplicable. But that there is a most striking analogy in the consecutive concatenation of morbid phenomena that characterize bilious fever and dysentery, I think no one conversant with these diseases will seriously dispute. There is the same functional aberration in both, and but for the muco-sanguineous dejections, and tenesmus, that so characteristically distinguish dysentery, it were *identical* with bilious fever.

Professor Calhoun, in his edition of Gregory's Practice, remarks, that "dysentery is generally a form of the bilious remittent fever of summer and autumn, and is produced generally by animal and vegetable decomposition; the effects of which are brought into action by the cold air of evening, sudden vicissitudes of temperature, or any cause which cools the surface and gives the disease a centripetal direction." The fact too that the symptoms of dysentery may obtain in all their virulency, viz: bloody and mucous stools, tormina and tenesmus, from other causes than those capacitated to develope idiopathic dysentery, shows, I think, pretty conclusively, that the disease as ordinarily exhibited, should be regarded as a mere adventitious symptom of bilious fever. It does seem to me if this view of dysentery were generally taken, that its treatment would be very much simplified, and its fearful mortality eminently abridged.

Although a most interesting enquiry, it must be conceded, that the time and labor spent in the sedulous investigation of the remote cause of disease, are, for the most part, unprofitable, for even were our researches rewarded by a revelation of the first link in the chain of morbid action, it would not at all induce to its removal. For admitting that malaria is the remote cause of dysentery, we know of no agent that would eliminate it from the economy, or render it innoxious; indeed we do not know what malaria is—so much was the judicious Sydenham impressed with the futility of this enquiry, that he remarks, "that the discovery and assigning of remote causes, which engross the thoughts and feed the vanity of curious enquirers, *is an impossible attempt;* and that only the immediate and conjunct causes fall within the compass of our knowledge, and that from *from them alone* the curative indications are to be taken."

My object is not so much to establish the remote cause of dysentery, considered as a distinct disease, as to prove its identity with that morbific agent, whatever it may be, that so modifies or changes the molecules of the nervous tissue as to set up a predisposition to bilious fever.

Of however little use, in general, the understanding aright of the remote cause may be, in suggesting the correct curative indications, still, in the disease under consideration, I regard it of importance, because otherwise we would not be enabled to so well understand the near relationship it bears to bilious fever. If we were to confine our inquiries to the pathological phenomena developed in autopsic inspection, without taking into contemplative consideration the consecutive morbid changes from the *aggression* to the *finale* of the disease, we should deduce an inference intolerant of the identity of dysentery and bilious fever, and the therapeutic medications suggested by these *data*, would be essentially different in the two diseases; and although they would prove successful in the latter, the physician, in a great majority of instances, would be chagrined and mortified by their total failure in the former, as all can testify who have been compelled to grapple year after year with this terrible malady.

I am aware that modern practice assumes to make the curative indications in disease harmonize with its pathological condition, as previously ascertained by *post mortem* examinations; and this, indeed, would seem the only just method of establishing a sound and philosophical therapeutics.

The ancient method of predicating a system of practice upon mere ideal and fanciful hypothesis, was certainly absurd and unsatisfactory, and must of necessity, not unfrequently, have led to mischievous and disastrous consequences. Yet, upon an honest and impartial inquiry and comparison, I apprehend that, notwithstanding a flood of light has of late years been shed upon the science of medicine, by researches into general and pathological anatomy, so far as ability to *cure the sick* is concerned, the moderns have not so much cause for self-gratulation as would at first appear. For the pathology of a disease, as developed by autopsic examination, must necessarily refer to that period which immediately preceded the dissolution of the subject; and it is at least not improbable that some of the morbid appearances were consummated *in articulo mortis.* The pathological condition cannot be ascertained at any curable period of the disease, and has, consequently, at last to be *inferred* from the appearances of an *incurable* period.

I have been induced to make these desultory remarks upon pathology, to show that the argument in favor of exclusively deducing therapeutic medications from appearances after death, is more specious than real, and must frequently lead to a most mischievous practice, as I hope more fully to show when I come to speak of the treatment of dysenteric bilious fever.

Having said this much, I feel inclined to go immediately into the consideration of the proper treatment of this disease, only stopping to premise some conjectures in reference to its probable pathology at a *curable* period, for I care but little about its pathology, so far as the treatment is concerned, when the subject of it is on the dissecting table. But as I set out with a view to be somewhat methodical in this dissertation, I shall proceed succinctly to detail the appearances after death in this disease, as I have repeatedly witnessed, and as noted by others.

Gregory tells us about the "appearances in severe and protracted cases." I regard any case as of reasonable severity when it affords the practitioner an opportunity for a *post mortem* examination. When dysentery is so "severe and protracted" as to destroy life, we find the mucous membrane lining the large intestines in a condition evincive of a precedaneous high state of inflammation, studded over with small grey or yellow tubercles, occasionally abraded, and sometimes ulcerated, at least some portion of it.* Somewhat elevated above the circumjacent tissue, red patches are to be met with, containing numerous small vesicles. These evidences of primordial phlogosis are, for the most part, confined to the large intestines; though it is sometimes the case that they may be met with throughout the intestinal tract; and Dr. Dewees mentions a case of "black vomit" in dysentery, which goes to show that the mucous membrane of the stomach may also be the seat of sthenic hyperæmia in this complaint.

Dr. Preston, in his observations on this disease, as it existed in Cymerie in 1821, remarks, that "the liver was invariably deeply engaged in the disease; it was, in general, greatly enlarged, and its whole structure apparently entirely destroyed." Dr. Eberle says, "the liver is always, perhaps, functionally deranged in this disease," but, unless where it occurs epidemically in "hot and insalubrious climates," there is, generally, no organic lesion of this viscus.

Having thus cursorily surveyed the more prominent points in the essential pathology of dysentery, to establish its identity with bilious fever, it will be convenient to institute a parallel between them, by a just comparison of their relative symptoms; from which I think it will appear, that, up to the intestinal manifestations, they are undistinguishable.

---

* Morgagni mentions one case of ulcerated intestinal mucous membrane only, but cites many others who declare they have seen it. Bayle, Cayol, Vaidy and Fournier esteem it rare. Broussais does not mention it. Dr. Cheyne regards it as common.

| SYMPTOMS OF BILIOUS FEVER. | SYMPTOMS OF DYSENTERY. |
|---|---|
| 1. Languor, drowsiness, slight chills, alternating with flushes of heat, until the latter predominates and a hot stage is established. | 1. It begins with a chilliness and shaking, immediately succeeded by a heat of the whole body as is usual in fevers.—*Sydenham.* |
| 2. Tongue covered with a brownish fur, nausea and occasionally bilious vomiting.—*Eberle.* | 2. Tongue coated with a brown fur along its middle, and sometimes nausea and bilious vomiting.— *Eberle.* |
| | Frequent and irresistible inclination to discharge the contents of the rectum, without a corresponding ability, the effort resulting merely in the expulsion of a little mucus, which may or may not be tinged with blood; or it may be that the discharge be blood entirely—the discharge preceded by tormina, or cutting pains, followed by tenesmus. |

It will be observed that the intestinal symptoms developed in the progress of dysentery, alone, have suggested its cognomen, and these alone have given it a specious claim to a separate existence upon the pages of nosology. That there is the same congestion in the portal circle, and derangement in the hepatic system generally, in both dysentery and bilious fever, is at this day, no matter of controversy among enlightened physicians; and the intestinal symptoms that so peculiarly characterize dysentery are, perhaps, legitimately referable to the nature of the exciting cause; which, acting in such manner as to occasion a sudden centripetal direction of the fluids, sets up a *peculiar* inflammation in the lining membrane of the intestines. Now, it is probable, had the same cause acted less energetically, a bilious fever, and not a dysentery, would have resulted.

It will follow, if my views in reference to the identity of bilious fever and dysentery be correct, that the curative indications, and therapeutic medications will be the same in both; save that shade of difference made necessary to meet the additional symptoms in the latter. It is an unflinching adherence to this theory and practice, that has enabled the author, for the last several years, to close hermetically, in his practice, this widening outlet of human life.

In 1831, the disease prevailed in this district with even unwonted violence, hurrying with demoniac ferocity large numbers from time to eternity. The physicians of the country, with myself, resorted to every expedient to arrest its mad career, or abate its fury, but still it swept onward with unmitigated violence. Fæcal evacuations induced by the exhibition of laxatives, ceased with their operation; and regardless of opiates, the supervention of agonizing tormina, bloody and mucous stools, with tenesmus, were inevitable—calomel in large doses, as advised in the epidemic dysentery of high latitudes, seemed but to hasten the catastrophe. A combination of sugar of lead, calomel and ipecac., followed by anodyne enemata, and preceded by copious blood-letting, failed in its promised beneficence—small doses of calomel, followed by castor oil and laudanum, procured but a temporary respite. Blood-letting, the warm bath, Dover's powder and calomel, to be followed by castor oil, added but another failure. This uniform want of success could not fail to produce feelings of a most unpleasant character, and the reflection that others were equally unsuccessful, (that soothing consolation to a little mind,) but added to the poignancy of the regret. Under this lamentable state of things, opportunities for autopsic inspection were sought with avidity, with a faint hope, that some new indication of cure might thence arise. I examined carefully the body of a negro, who had died suddenly from the disease. The usual morbid appearances were present—congestion of the portal circle with a manifest colonitis. The cells of the colon contained a quantity of hardened fæces, covered with a tenacious, glassy mucus, which seemed to confine these scybalæ to the cells. The tormina and tenesmus, in this case, had been distressing beyond conception, and death seemed to result, rather from the violence of the pain, than the termination of the disease. In my cogitations upon this case, the inquiries arose in mind, may not this condition of the colon be generally present in dysentery? may not the fetid, and *cider colored* discharges, that occur involuntarily just before death, be a solution of these fæces induced by a relaxation in *articulo mortis* and a consequent increased flow of fluid into the bowels and which may account for the empty condition of the colon generally in *post mortem* examinations. Again—medicines that possess a relevancy of action for the small intestines, would not be calculated to dislodge these fæces, but would force the contents of the bowels from above, through the centre of the colon, leaving them *impacted* in the cells on either side.

It is not my desire here to essay to revive the antiquated and obsolete doctrine, that scybalæ are the *cause* of dysentery, for I do not believe it. But I do believe that they, or other accumulations in the colon, are the cause of *death*, and deem it of primary importance that they should be removed. I would not, to be sure, be so infatuated as to continue the purging till these scybalæ were produced in a *tangible* shape, which I believe was the practice formerly in vogue, it being altogether sufficient for my purpose, to continue it until tenesmus and bloody and mucous stools subside.

The foregoing considerations induce me to propose as the proper *methodus curandi* in dysentery,

1. To moderate inordinate action of the heart and arteries.
2. To remove the engorgement of the liver and portal circle.
3. To remove the irritating fæces collected in the cells of the colon.

4. To restore the healthy action of the cutaneous exhalents, by giving a centrifugal direction to the fluids.

The first and fourth indications, are to be fulfilled by blood-letting, nauseants and ablutions with some detergent material—weak, warm ley answers a valuable purpose.

The second and third indication are to be fulfilled, by a combination of calomel, rhubarb and *aloes.* The aloes being an essential ingredient, from its known action upon the large intestines.

Aloes, although under circumstances ordinarily concomitant with hyperæmia of the mucous tissue of the large intestines, is contra-indicated, yet, if my views of dysentery be correct, it follows that it is a *sine qua non*, and with a just conception of the hazard and responsibility of aberrating from the beaten track, and going counter to all authority, I am constrained by a confidence in the article, that the experience of several years has served to strengthen and fortify, to insist upon its exhibition, to fulfil an indication of paramount importance, *that no other agent in the materia medica can fulfil.* A combination of aloes, rhubarb and calomel, is peculiarly felicitous in acting beneficently upon the whole intestinal tract, and simultaneously dispersing congestion of the portal circle, and imparting to the liver a renovated functional energy. It is therefore fitted with peculiar adaptation to dysentery, and whoever exhibits it once in this disease, will not be easily tempted by a specious, and sophisticated ratiocination, to abandon it. The castor oil so frequently advised by authors, and prescribed with so much confidence by physicians, is most unsatisfactory, and I believe mischievous in its operation. From its unctuous and oleaginous quality, it is supposed to glide soothingly over the inflamed membrane, dislodging, and at the same time, defending it against acrimonious humors. This is a great mistake, attended not unfrequently with serious consequences. To prove this, let castor oil be applied as a dressing after vesication by cantharides, when great pain and inflammation will result—applied to a common cut it produces much pain, swelling and inflammation; thus demonstrating its acrimonious character. It were scarcely unfair to trace the seeming intractability of the disease, in many cases, to the frequent exhibitions of this article. It is the exquisite irritability produced by castor oil, that has led those who employ it, into an extravagant praise of opium; so urgent are the tormina, and tenesmus, after the operation of a dose of castor oil, as to demand imperatively a large dose of opium, to secure the patient a temporary respite from intense agony. In the plan of treatment here proposed, castor oil, with all its class, collocated under the head of laxatives, is I think justly, proscribed as nugatory or injurious. It is true they might be made available in cases so slight as to demand but little medical interference; but I hold it to be a good maxim in physic, never to tamper with inefficient means, when we have at command those upon which we can confidently rely.

When called to treat a case of dysentery, if the subject be stout, with a strong, full pulse, I never hesitate to bleed him, more or less, but *always* to an amount that will insure a reduction in the force and frequency of the pulse. If the tongue be coated, with a bitter taste in the mouth, and especially if there be occasional bilious vomiting, an emetic of ipecac. follows the bleeding—so soon as the stomach becomes composed after the emetic effort, I direct that four of Cooke's pills be taken, which failing to operate in six hours, one more is to be taken every hour, till fæcal evacuations are produced. After three or four operations are produced by the pills, I administer two tea-spoonsful of the camph. tinct. opii to quiet the bowels, and stop any further cathartic effect of the pills—so soon as the dysenteric discharges return, I direct that one of Cooke's pills be taken every hour, till they operate; and if there be much fever, or a dry skin, I prescribe the following powder, to be taken alternately with the pill, till nausea be produced:

℞ Ipecac. gr. ss.
Nit. potas. gr. v.
Cr. tart. gr. x. M.

The operation of the pills to be followed, as in the first instance, by the camph. tinct. opii.

If mucus, or muco-sanguineous discharges again make their appearance, *which I have never yet seen*, the same routine should be enforced. The tenesmus and tormina, by this plan of treatment, are disarmed as if by enchantment, and the recovery *of every case* (and they have been numerous,) in which I have called it into requisition, has been most rapid.

As the thirst is very urgent in this affection, and as cold water, so much craved by the patient, is almost certain to excite griping, I direct him to drink common table tea, made palatable with cream and sugar, or sage, or balm, if he prefer it, with the privilege of returning to cold water, whenever it fails to excite griping. The diet of course should be meagre, consisting of rice-water, thin gruel, sago or tapioca.

When the accompanying fever is of the intermittent type, one grain of the sulphate of quinine is ordered every hour, till twelve grains are taken during the apyrexia, regardless of the dysenteric symptoms. The quinine to be followed by Cooke's pill.

In children too small to take the pills, the same compound is exhibited in powder, the dose being graduated to the age of the patient. It is absolutely astonishing, with what facility and certainty these powders, with nothing else, save a few drops of paregoric after their operation, will entirely remove this complaint in small children. Where the little sufferers have become low from neglect or mismanagement, I have derived the greatest benefit from washing their whole bodies well, with warm weak ley, drying them carefully, and putting them to bed, before any medicine is exhibited.

I have had, for the last four years, frequent opportunities of testing the efficacy of this practice, and can say, with the most rigid adherence to truth, that I have never lost a patient with dysentery since I adopted it; which was in the very height of one of the most afflictive and fatal visitations ever known in this region.

*February* 21, 1840.

